# Androgens and the masculinization programming window: human–rodent differences

**DOI:** 10.1042/BST20200200

**Published:** 2020-08-11

**Authors:** Richard M. Sharpe

**Affiliations:** MRC Centre for Reproductive Health, The Queen's Medical Research Institute, University of Edinburgh, 47 Little France Crescent, Edinburgh EH16 4TJ, U.K.

**Keywords:** androgens, backdoor pathway, fetal programming, masculinization, placenta, testicular dysgenesis syndrome

## Abstract

Human male reproductive disorders are common and may have a fetal origin — the testicular dysgenesis syndrome (TDS) hypothesis. In rats, experimentally induced TDS disorders result from disruption of fetal androgen production/action specifically in the masculinization programming window (MPW). MPW androgen action also programs longer anogenital distance (AGD) in male versus female rats; shorter male AGD is correlated with risk and severity of induced TDS disorders. AGD thus provides a lifelong, calibrated readout of MPW androgen exposure and predicts likelihood of reproductive dysfunction. Pregnant rat exposure to environmental chemicals, notably certain phthalates (e.g. diethyl hexl phthalate, DEHP; dibutyl phthalate, DBP), pesticides or paracetamol, can reduce fetal testis testosterone and AGD and induce TDS disorders, provided exposure includes the MPW. In humans, AGD is longer in males than females and the presumptive MPW is 8–14 weeks’ gestation. Some, but not all, epidemiological studies of maternal DEHP (or pesticides) exposure reported shorter AGD in sons, but this occurred at DEHP exposure levels several thousand-fold lower than are effective in rats. In fetal human testis culture/xenografts, DEHP/DBP do not reduce testosterone production, whereas therapeutic paracetamol exposure does. In humans, androgen production in the MPW is controlled differently (human chorionic gonadotrophin-driven) than in rats (paracrine controlled), and other organs (placenta, liver, adrenals) contribute to MPW androgens, essential for normal masculinization, via the ‘backdoor pathway’. Consequently, early placental dysfunction, which is affected by maternal lifestyle and diet, and maternal painkiller use, may be more important than environmental chemical exposures in the origin of TDS in humans.

## Introduction

Reproductive disorders in newborn boys (cryptorchidism, hypospadias) and in young men (low sperm count, testicular germ cell cancer) are remarkably common, are inter-related, are probably increasing in incidence [1,2] and point to increasing male/couple infertility [1,3]. These clinical observations led to the concept of a testicular dysgenesis syndrome (TDS), which proposed a common fetal origin for these disorders [1,4], probably involving impaired androgen production by the fetal testis [5,6]. However, as it is impossible to dynamically monitor fetal testis development or to routinely measure fetal androgen production during human pregnancy, the TDS concept remained largely hypothetical, despite strong circumstantial supporting evidence [1].

## The ‘masculinization programming window’ (MPW) and importance of anogenital distance (AGD)

A radical change in thinking occurred with the discovery in 2008 in laboratory rats of the so-called MPW [7]. This demonstrated that normal reproductive differentiation and development in fetal males is absolutely dependent on being exposed to a sufficient level of androgens during a critical fetal period, the MPW, which in rats is just after testis differentiation, within the embryonic(e) period e15.5–e18.5 [6,7]. If androgen exposure during the MPW is lowered experimentally in rats, a TDS-like spectrum of reproductive disorders becomes manifest at birth and/or in adulthood, the severity of the disorders being linked to the degree of androgen blockade [7] or androgen suppression in the MPW [6,8]. In contrast, comparable androgen suppression in fetal life immediately after the MPW (e.g. from e19.5–e21.5 in rats) has none of these adverse effects [6]. One of the pivotal findings to emerge from these studies was that AGD, which is normally about twice as long in male as in female rodents postnatally [9], provides a life-long readout of the level of androgen exposure specifically within the MPW [6,7]. The male–female difference in AGD has been used for decades by toxicologists as an indicator of the normality of fetal reproductive development in male rodents [9] and is how animal technicians determine sex of laboratory animals at birth. However, the critical importance of the MPW androgen exposure–AGD relationship is that it has enabled translation of rodent experimental studies to clinical observational studies in humans [6,9,10].

**Figure 1.**
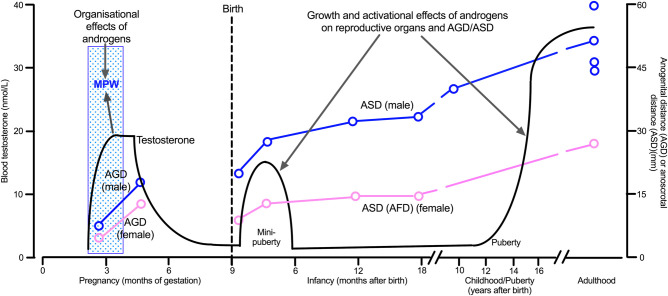
The different roles of androgens in the human male during the presumptive fetal masculinization programming window (MPW; organizational/programing effects) and during postnatal life (mini-puberty and puberty; growth and activational effects), in relation to average blood testosterone levels (solid black line). Anogenital distance (AGD; fetal life) or anoscrotal distance (ASD; postnatal life) is shown to illustrate how androgen exposure in the MPW programs longer AGD/ASD in males than in females, a change evident already in mid-pregnancy; in females the equivalent measurement to ASD is from the center of the anus to the fourchette (AFD). Note that the absolute length of ASD increases postnatally under the influence of postnatal androgens, primarily during mini-puberty. ASD rather than AGD is shown for postnatal males as this is the measurement most commonly used in epidemiological studies, but comparable sex differences are evident in postnatal AGD as for ASD, except AGD is much longer than ASD (e.g. in adult males AGD = 121–140 mm versus 44–60 mm for ASD). Direct measurement of AGD is only available between 11 and 18 weeks’ pregnancy but measurement by ultrasound is available later in pregnancy (see text) but is not illustrated. Note that for adult males, mean values for ASD are illustrated from four independent studies to give an idea of the variation in adult male ASD. The illustration is based on data from the literature [11–14,30–33,37] with childhood data based on mean reference data obtained using the Cambridge measurement method [12].

As in rodents, human males have a longer AGD than females at birth/during childhood and adulthood [10–14] (Figure 1). This sex difference first emerges at 11–13 weeks’ gestation and becomes maximally different (1.7- to 2-fold) by 17–20 weeks’ gestation, whether measured directly in abortuses [15,16] or *in utero* by ultrasound [17–21]. Thus, if the male–female difference in AGD is determined in humans in a MPW, as it is in rodents, this clearly locates the MPW in the 1st trimester, probably within the period 8–14 weeks’ gestation [7,10] (Figure 1). The (indirect) evidence to support this interpretation is quite strong, because in humans the occurrence of TDS disorders is associated with lower AGD, whether for hypospadias [22–26] and cryptorchidism [23,27,28] around birth or for testis size and sperm count and semen quality in adulthood [29–33]. These findings are analogous to results from experimental studies in rodents showing that lower AGD and TDS disorders arise specifically because of reduced androgen exposure in the MPW [6,7].

## Androgen exposure in the MPW and requirement for postnatal androgen exposure

From the foregoing discussion it is reasonable to conclude that, in humans, the level of exposure to androgens in the presumptive MPW plays a fundamental role in determining later development and function of the male reproductive tract, as it does in rodents (Figure 1). Moreover, AGD provides a life-long readout of the level of androgen exposure in the MPW. Thus in theory, AGD at birth or in early childhood, for which there is now robust reference data [12], might be able to forecast the likelihood of adult reproductive dysfunction. However, there are important caveats to this line of thinking.

First, standardized measurement of AGD is more difficult in humans than in rodents, and anoscrotal distance (ASD) rather than anogenital distance (AGD) is the currently preferred measurement postnatally (discussed in more detail in refs. [9,10,12,34]) (Figure 1). Second, although MPW exposure to androgens may ‘programme’ the final size of AGD and the reproductive organs, further exposure to androgens in postnatal life is required to grow AGD and the reproductive organs in adulthood to their pre-programmed size (Figure 1). Normally, this is achieved via testicular androgen production during mini-puberty and puberty [35]. However, if there is a deficiency in postnatal androgen production, for example because of hypogonadotrophic hypogonadism [36], then the pre-programmed ceiling of reproductive growth may not be achieved. This is illustrated clearly in rodents that lack a functioning hypothalamic-pituitary (HP) axis [37]. In such animals, AGD at birth is that of a normal male [38,39] because androgen production by the fetal testis in rodents during the MPW is paracrine-regulated and does not require a functional HP-axis [37], as detailed further below. However, because of the absence of a normally functioning HP-axis after birth, testicular androgen production is curtailed. Consequently, the androgen-dependent growth of AGD and reproductive organs that normally occurs postnatally (Figure 1) does not happen, and there is failure of puberty and consequent infertility [38,39]. If normal postnatal androgen exposure is restored experimentally in rodents without a functioning HP-axis, AGD and testis size are stimulated to grow to normal adult size [38] because they had been exposed to normal androgen levels during the MPW [40,41]. In human males with hypogonadotrophic hypogonadism, a similar defect in postnatal androgen-dependent growth of AGD and reproductive organs would be likely. In theory, this defect should be corrected by appropriate treatment of the hypogonadism, although whether this requires treatment during mini-puberty (which is rarely done — see refs. [42,43]) and/or puberty is unclear [36,42]. Nevertheless, what this example illustrates is that normal male reproductive development requires both ‘organizational’ exposure to androgens (in the MPW) followed postnatally by ‘activational’ exposure to androgens (during mini-puberty, puberty) (Figure 1). Deficiencies in the former are non-correctable (hence TDS disorders), whereas activational exposure to androgens is, in principle, correctable by treatment to normalize LH and testosterone secretion [36,42,43].

## Androgen exposure in the MPW and its potential disruption in rats

**Figure 2.**
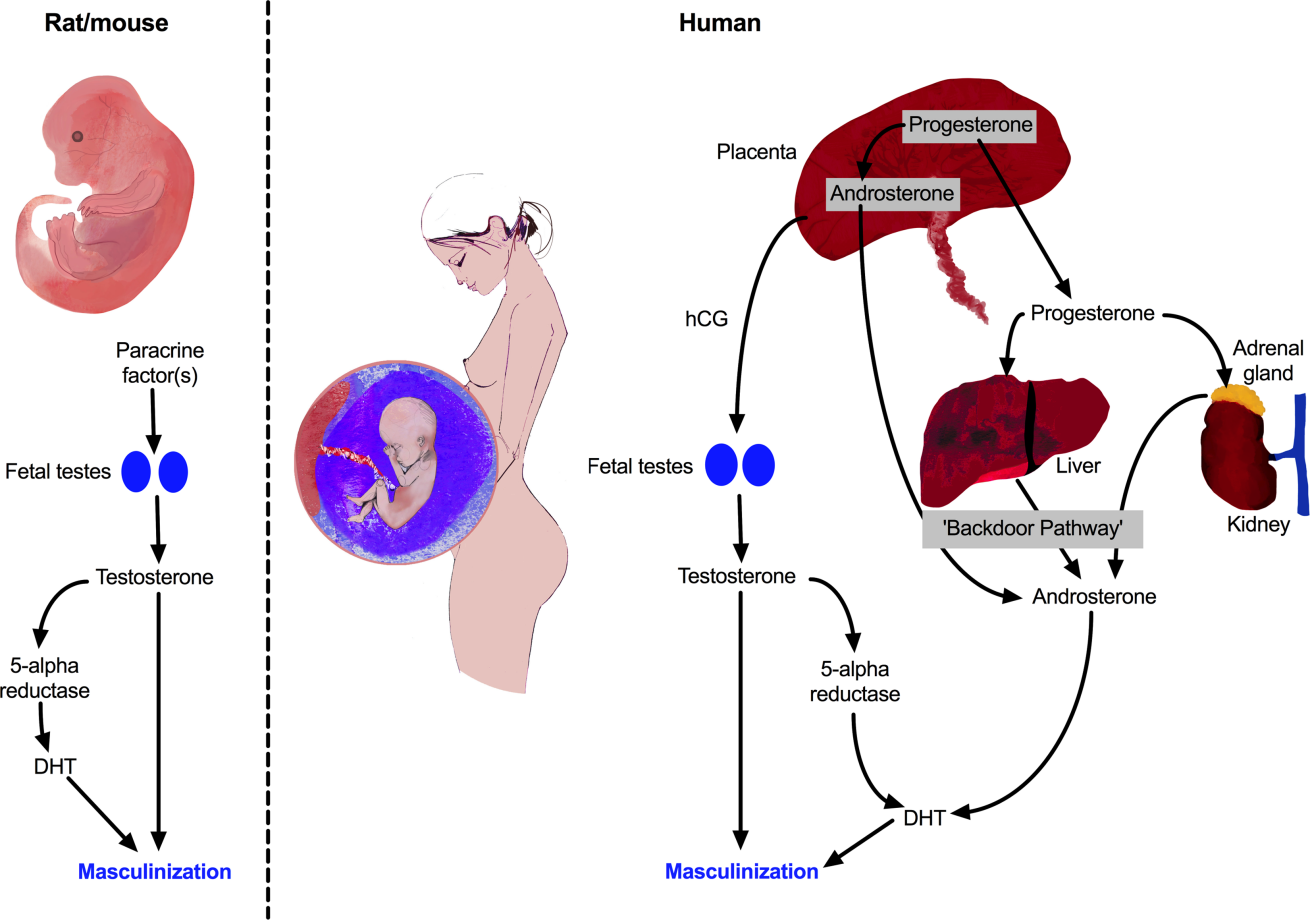
Human–rodent differences in the regulation of testosterone and dihydrotestosterone (DHT) production during the masculinization programming window. In rodents, masculinization is totally dependent on testosterone secretion by the fetal testes, which is under local (paracrine) control. In humans the set-up is more complex and there are two fundamental differences from rodents. First, testosterone production by the fetal testes is driven primarily by human chorionic gonadotrophin (hCG) produced by the placenta. Second, several fetal organs (placenta, liver, adrenals) in addition to the fetal testes, contribute to the androgen production that drives masculinization via the so-called ‘backdoor pathway’ [94,95].

The critical issue for masculinization of males is what factors determine or influence androgen exposure during the MPW. In rodents, the key event is the level of androgen production (primarily testosterone) by the fetal testis, as anything that perturbs this function during the MPW is likely to reduce AGD and ultimate reproductive organ size/function [6,10,40,41]. However, the activity of 5α-reductase enzymes which convert testosterone to the more potent dihydrotestosterone (DHT) in androgen-target reproductive tissues such as the genital tubercle (penis) and prostate is also important [44]. Additionally, conversion of testosterone to oestradiol may also play a role in penile development [45]. Nevertheless, these processes which occur in reproductive target organs are completely dependent on the provision of substrate (testosterone), which is produced by the fetal testis (Figure 2). Thus, the key question in rodents is what sorts of factors or exposures can interfere with normal testosterone production by the fetal testis during the MPW? Numerous studies have addressed this and have shown that a range of environmental chemicals can suppress fetal testis testosterone production [8,9,37]. Most important of these are certain phthalate esters, for example diethyl hexyl phthalate (DEHP) and dibutyl phthalate (DBP), as these are ubiquitous in our environment and the vast majority of humans are exposed chronically to them [46,47]. Maternal exposure of rats to such phthalates dose-dependently suppresses testosterone production by the fetal testis [8,9,48], and when exposure includes the MPW it induces a TDS-like syndrome in the resulting male offspring [6].

Various pesticides or fungicides (e.g. vinclozolin, prochloraz, linuron) can also inhibit fetal testis testosterone production dose-dependently in rats [8,9] as can exposure to common painkillers such as paracetamol (acetaminophen) or ibuprofen at human therapeutic levels [49,50]. Of particular importance and concern is that mixtures of these environmental (phthalates, pesticides) and/or pharmaceutical compounds can have additive effects in suppressing fetal testis testosterone, reducing AGD and inducing consequent TDS disorders [51–53]. Perhaps the most critical observation in these chemical mixture studies is that even when all of the individual compounds are present at doses substantially below their individual no-effect levels, there are profound adverse reproductive developmental effects in males exposed to such mixtures *in utero* [53].

## Androgen exposure in the MPW and its potential disruption in humans

The findings described in rats above have prompted numerous epidemiological studies in humans, examining whether exposure to any of the ‘anti-androgenic’ chemicals during pregnancy is associated with altered AGD or occurrence of hypospadias or cryptorchidism at birth in sons. Results from these epidemiological studies are quite mixed and are too complex and numerous to discuss in detail here (for systematic reviews see refs. [47,54–57]). For example, although several studies have shown a significant association between maternal exposure to DEHP or certain other phthalates in pregnancy and reduced AGD in sons at birth (e.g. [58,59]), other comparable studies have not confirmed this [47,57,60,61]. However, the consensus of meta-analyses is that DEHP exposure in pregnancy probably is associated with a small reduction in AGD in resulting sons [47,56,57]. Similar mixed results have been reported for maternal exposure to pesticides and AGD in sons [55] or perfluroalkyl substances (PFAs) [62,63]. There is more agreement that painkiller (e.g. paracetamol) exposure at human therapeutic levels can reduce fetal testis testosterone in both rodents and humans under experimental conditions [49,64] and maternal paracetamol exposure is associated with reduced AGD in resulting sons [65,66].

A particular issue with the association studies involving exposure to phthalates or pesticides, but not painkillers, is that the doses of individual compounds that induce effects via *in utero* exposure in rats are generally far higher than the levels of exposure that occur in the epidemiological studies [56]. For example, for the phthalate DEHP, extensive biomonitoring studies have shown that exposure in the general population is in the range <1–25 μg/kg/day [67], and in studies showing reduced AGD in boys at birth in association with maternal DEHP exposure, exposure has been mostly within this range. In contrast, in rats only maternal exposure to doses of 50–100 mg/kg/day and higher induce reductions in AGD in resulting male offspring [56,68,69], although one study reported a small but statistically significant reduction at 10 mg/kg/day DEHP [70]. Even higher exposure to DEHP or DBP (250–750 mg/kg/day) is required to induce TDS disorders such as hypospadias and cryptorchidism in rats [71,72]. Differences in metabolic rate between rodents and humans and potentially slower metabolism of phthalates in the fetal compartment have been suggested as ways of reconciling these rodent-human exposure differences [56], but this still does not close the gap and is based on limited data and several assumptions.

An alternative way to reconcile the dose disparity for rat-human findings is to assume that the human fetal testis is several thousand-fold more sensitive than the rat to DEHP and DBP in terms of their ability to suppress testosterone production. However, when fetal human testes are exposed to DEHP (or DBP) at doses equivalent to 500 mg/kg/day in rats, either in *in vitro* culture or as fully functioning *ex vivo* xenografts in immune compromised mice, there is no suppression of testosterone production, whereas there is in the rat under equivalent conditions [73–76]. As these are artificial experimental systems they cannot exclude the possibility that fetal human testis steroidogenesis *in utero* might be compromised by DEHP and DBP exposure. However, this also means accepting that the human fetal testis is hugely more sensitive to these chemicals than is the rat, which is the opposite of the direct evidence [64,77]. Moreover, two other pieces of human/primate *in vivo* evidence do not support this hypothesis. First, some women with inflammatory bowel disease (IBD) have been exposed to 50–100 times more DBP than the normal population because the pharmaceutical (5-aminosalicylic acid) which they took to control their IBD, including throughout pregnancy, had an enteric coating containing DBP in some cases [78]. Studies of the outcomes of pregnancy in these women has not reported any increase in masculinization disorders in resulting sons [79]. Second, *in utero* exposure of pregnant marmoset monkeys to 500 mg/kg/day monobutyl phthalate (the active metabolite of DBP) during the period of the presumptive MPW did not induce any reproductive disorders in the resulting male offspring as it would in rats [80]. Finally, it should be noted that testosterone production by the fetal mouse testis, like the human, is also unaffected by exposure to high doses of DEHP or DBP whether *in vitro* or *in utero* [64,77].

## Human–rodent differences in control of androgen production during the MPW

Perhaps the biggest concern in extrapolating rat (or mouse) fetal testis testosterone effects to the human is that the regulation (and importance) of testosterone production by the fetal testis is fundamentally different in the rat and human (Figure 2). Remarkably, this difference is rarely mentioned in the literature on fetal testosterone effects. As noted above, normal testosterone production by the rat and mouse fetal testis during the MPW does not require either functional LH receptors or LH, although the paracrine factors that actually drive steroidogenesis at this time remain unclear [37]. In contrast, normal testosterone production by the human fetal testis requires both functional LH receptors and hCG (human chorionic gonadotrophin), which is an LH-like analog produced by the placenta in humans and in non-human primates [37,81]. Thus, the driver for testosterone production by the human fetal testis during the MPW is fundamentally different from that in the rat and mouse (Figure 2). However, it cannot be excluded that some degree of testosterone production might be hCG/LH receptor-independent in the human, early in the MPW [37]. Nevertheless, if a fully functioning LH receptor is not present, due to a coding mutation, then the human male fetus fails to normally masculinize by birth, indicating subnormal androgen exposure in the presumptive MPW, whereas comparable inactivating mutations in mice have no effect on masculinization (reviewed in [37]). After birth in both humans and rodents, inactivating LH receptor mutations or mutations in LH or in upstream pathways that impair pituitary LH production, impair male reproductive development (e.g. testicular and penile growth) due to effects on postnatal testosterone production (i.e. ‘growth and activational’ effects in Figure 1) [36,38,39,41,81].

An important consequence of the LH receptor-dependence of testosterone production by the human fetal testis during the presumptive MPW is that this also means there is parallel dependence on normal hCG exposure (Figure 2), because without this ligand normal testosterone production would not occur [37]. It is generally presumed that hCG production by the normal placenta is in excess of that needed to stimulate testosterone production by the 1st trimester fetal testis. However, it is well established that low birthweight is a major risk factor for hypospadias and cryptorchidism [82,83], in particular, impaired placental development and function in the 1st trimester with resulting fetal growth restriction [84–86]. Indeed, it has been estimated that nearly 1 in 5 boys born after fetal growth restriction will present with hypospadias [85]. This leads to the scientifically appealing notion that 1st trimester placental dysfunction might lead to reduced hCG production and thus to reduced testosterone production by the fetal testis during the MPW. Unfortunately, this hypothesis is not supported by the available data which points, if anything, to increased hCG production in such cases [87,88]. However, it is possible that placental dysfunction might impair the production of factors other than hCG, for example progesterone, that are needed for normal androgen production in human fetal males (Figure 2). This possibility leads on to another fundamental difference between humans and rodents as discussed next.

Direct information about the fetal masculinization process in the 1st trimester in human males is obviously limited and most insight has come from cases of masculinization disorders. Such disorders have been associated with specific gene mutations, most of which impinge either on the sexual differentiation process [89] or on testicular steroidogenesis [37,90]. However, a surprise finding to have emerged has been identification of the so-called ‘backdoor pathway’ for synthesis of DHT and demonstration that inactivating mutations in the steroidogenic enzyme genes involved in this pathway are associated with masculinization disorders [91]. The latest findings show that the backdoor pathway operates minimally within the human fetal testis but points instead to interactions between the placenta, liver and adrenal glands as organs within which the component enzymes of the backdoor pathway have the greatest activity [92]. Based on these findings, the authors suggest that placental progesterone may provide the basic substrate which is then converted to androsterone in the placenta, liver and adrenal gland, which in turn is converted to DHT within the genital tubercle and other DHT target organs (e.g. prostate) (Figure 2). In other words, androgen-driven masculinization of the reproductive tract in human males is functionally multi-organ dependent rather than just testis-dependent as in rodents (Figure 2). This may also help to explain the strong association that exists between early placental dysfunction and greatly increased risk of cryptorchidism and hypospadias [82,83,92], as discussed earlier. This is possibly related to altered progesterone production by the placenta [93]. However, our understanding of the functional dynamics of the backdoor pathway during the 1st trimester and what non-genetic factors might perturb it are superficial, and it remains unknown how this relates to fetal testicular function at the time. In this regard, it is emphasized that steroidogenic function of the testis during the presumptive MPW is still considered to be the main player in masculinization of the human male fetus.

## Concluding remarks and future perspectives

It has long been accepted that normal masculinization of the fetus is absolutely dependent on testosterone produced by the fetal testis — and now we know that this probably has to occur during a specific period, the MPW. This understanding has derived primarily from experimental studies in rodents, because direct information is difficult to obtain for the human. Hopefully this brief review has shown that whilst the rodent experimental evidence appears highly relevant to man, there are fundamental differences between man and rodents that limit its seamless translatability. Chief among these is the basic difference in regulation of testicular steroidogenesis during the presumptive MPW in man (hCG–LH receptor-driven) versus rodents (paracrine-driven, LH receptor-independent), but other less crystallized differences may be functionally more important. For example, the fact that androgen production in the presumptive MPW in human males is doubly dependent on placental function, via hCG (testis effects) and progesterone (backdoor pathway effects) production, but perhaps also via other presently unknown pathways. These pathways are clearly important as placental dysfunction in early pregnancy is unequivocally associated with greater risk of masculinization disorders (hypospadias, cryptorchidism) in human males [82–86].

An important implication of the experimental animal–human differences is that the maternal lifestyle or environmental factors that might negatively impact androgen exposure during the MPW in humans could be fundamentally different from what does this under experimental conditions in rodents. So far, most human (mainly epidemiological) studies have simply followed a similar path to rodent experimental studies, focusing on environmental chemicals such as phthalates and pesticides, when searching for potential causes of impaired androgen exposure during the MPW. However, perhaps this is looking under the wrong lamp post. Arguably, more effort should be directed at identifying what external factors might impair early placental development and function, especially maternal diet and lifestyle which are known to be influential in this regard [94] (Figure 2), and which are modifiable. This does not exclude the possibility that environmental and lifestyle chemical exposures might play a role in disrupting androgen production in the MPW in humans, especially when considering additive effects of environmental and pharmaceutical compounds [53,95]. Nevertheless, in view of the profound differences in susceptibility between the rodent and human fetal testis to such disruption [65,77], as outlined in this review, we should not assume that specific environmental chemical effects reported in rodents are human-relevant unless there is direct supporting evidence (e.g. [95]). Furthermore, the focus of the ‘masculinization disorders’ field needs to shift to embrace non-environmental chemical exposures, such as those resulting from dietary and lifestyle choices and from pharmaceutical use in pregnancy (e.g. painkillers), and to take account of the central importance of placental function in governing normal androgen exposure in the MPW.

## Perspectives

- *Importance of the field*: Approximately 1 in 6 young men has a reproductive disorder, especially a low sperm count, and growing clinical and experimental evidence points to these disorders comprising a TDS that may be linked to deficient androgen exposure during early fetal life. Identification of the fetal MPW, provides a focus for research aimed at identifying modifiable factors in early pregnancy that have potential to disrupt androgen production or action during the MPW leading to TDS.
- *Summary of the current thinking*: Sufficient androgen exposure in the MPW is essential for programming normal masculinization and later reproductive tract/organ development, though normal androgen exposure postnatally (mini-puberty, puberty) is also important to fulfill this programming. Inappropriate exposure to certain environmental chemicals (e.g. phthalates, pesticides) in pregnancy has been the main focus of animal and human epidemiological research to identify maternal factors that might perturb androgen production in the MPW. However, direct studies of chemical effects on human fetal testis testosterone production raise questionmarks about the importance of these effects *in utero*.
- *Comment on future directions*: Regulation of androgen production in the human fetal male during the presumptive MPW differs fundamentally from that in rodent experimental models, and highlights the importance of normal placental development and function for normal androgen exposure. Future studies need to take more account of this, and of maternal dietary and lifestyle factors that are known to impact early placental development, although further evaluation of exposure to pharmaceuticals (e.g. paracetamol) alone or in combination with environmental chemical mixtures, and their effects on fetal testis function may also prove important.

## Open Access

Open access for this article was enabled by the participation of University of Edinburgh in an all-inclusive *Read & Publish* pilot with Portland Press and the Biochemical Society under a transformative agreement with JISC.

## Abbreviations

AGD: anogenital distance
DBP: dibutyl phthalate
DEHP: diethyl hexyl phthalate
DHT: dihydrotestosterone
hCG: human chorionic gonadotrophin
HP: hypothalamic-pituitary
IBD: inflammatory bowel disease
MPW: masculinization programming window
TDS: testicular dysgenesis syndrome
